# Differential Gene Expression in Thrombomodulin (TM; CD141)^+^ and TM^−^ Dendritic Cell Subsets

**DOI:** 10.1371/journal.pone.0072392

**Published:** 2013-08-23

**Authors:** Masaaki Toda, Zhifei Shao, Ken D. Yamaguchi, Takehiro Takagi, Corina N. D’Alessandro-Gabazza, Osamu Taguchi, Hugh Salamon, Lawrence L. K. Leung, Esteban C. Gabazza, John Morser

**Affiliations:** 1 Department of Immunology, Mie University Graduate School of Medicine, Tsu Shi, Mie Ken, Japan; 2 Stanford University School of Medicine, Division of Hematology, Stanford, California, United States of America; 3 Veterans Administration Palo Alto Health Care System, Palo Alto, California, United States of America; 4 Knowledge Synthesis Inc., Berkeley, California, United States of America; 5 Department of Pulmonary and Critical Medicine, Mie University Graduate School of Medicine, Tsu Shi, Mie Ken, Japan; National Cerebral and Cardiovascular Center, Japan

## Abstract

Previously we have shown in a mouse model of bronchial asthma that thrombomodulin can convert immunogenic conventional dendritic cells into tolerogenic dendritic cells while inducing its own expression on their cell surface. Thrombomodulin^+^ dendritic cells are tolerogenic while thrombomodulin^−^ dendritic cells are pro-inflammatory and immunogenic. Here we hypothesized that thrombomodulin treatment of dendritic cells would modulate inflammatory gene expression. Murine bone marrow-derived dendritic cells were treated with soluble thrombomodulin and expression of surface markers was determined. Treatment with thrombomodulin reduces the expression of maturation markers and increases the expression of TM on the DC surface. Thrombomodulin treated and control dendritic cells were sorted into thrombomodulin^+^ and thrombomodulin^−^ dendritic cells before their mRNA was analyzed by microarray. mRNAs encoding pro-inflammatory genes and dendritic cells maturation markers were reduced while expression of cell cycle genes were increased in thrombomodulin-treated and thrombomodulin^+^ dendritic cells compared to control dendritic cells and thrombomodulin^−^ dendritic cells. Thrombomodulin-treated and thrombomodulin^+^ dendritic cells had higher expression of 15-lipoxygenase suggesting increased synthesis of lipoxins. Thrombomodulin^+^ dendritic cells produced more lipoxins than thrombomodulin^−^ dendritic cells, as measured by ELISA, confirming that this pathway was upregulated. There was more phosphorylation of several cell cycle kinases in thrombomodulin^+^ dendritic cells while phosphorylation of kinases involved with pro-inflammatory cytokine signaling was reduced. Cultures of thrombomodulin^+^ dendritic cells contained more cells actively dividing than those of thrombomodulin^−^ dendritic cells. Production of IL-10 is increased in thrombomodulin^+^ dendritic cells. Antagonism of IL-10 with a neutralizing antibody inhibited the effects of thrombomodulin treatment of dendritic cells suggesting a mechanistic role for IL-10. The surface of thrombomodulin^+^ dendritic cells supported activation of protein C and procarboxypeptidase B2 in a thrombomodulin-dependent manner. Thus thrombomodulin treatment increases the number of thrombomodulin^+^ dendritic cells, which have significantly altered gene expression compared to thrombomodulin^−^ dendritic cells in key immune function pathways.

## Introduction

Thrombomodulin (TM, also known as fetomodulin, CD141 and BDCA3) was originally discovered as an endothelial cell surface protein that binds thrombin leading to a remarkable alteration of thrombin’s substrate specificity from pro-coagulant and pro-inflammatory to anti-coagulant and anti-inflammatory [1]. TM is composed of a C-terminal cytoplasmic domain, a trans-membrane domain and three extracellular domains consisting of a C-type lectin domain at the N-terminus, 6 copies of epidermal-growth factor-like (EGF) motifs and an O-linked domain [2], [3]. When thrombin binds to the EGF repeats of TM, cleavage of its pro-coagulant and pro-inflammatory substrates such as fibrinogen and protease activated receptor 1 are inhibited and activation of protein C (PC) to activated protein C (aPC) and procarboxypeptidase B2 (proCPB2, also known as thrombin activatable fibrinolysis inhibitor, TAFI, or procarboxypeptidase U) to CPB2 is increased [4], [5]. CPB2 is both an anti-fibrinolytic and anti-inflammatory metalloprotease while aPC is a serine protease possessing both anti-coagulant and anti-inflammatory activities [6], [7].

The lectin domain has been shown to be involved in inflammation by studies in mice that express TM without the lectin domain [8], [9], [10]. Recently we showed that treatment of mouse bone marrow-derived dendritic cells (DCs) with either soluble or cell-bound TM induced TM expression on their cell surface and that this effect was mediated by the lectin domain [11]. Levels of maturation markers such as MHC II as well as co-presentation molecules such as CD80, CD83 and CD86 were reduced. The TM^+^ DCs were tolerogenic when compared in adoptive transfer experiments in a mouse model of airway hypersensitivity to TM^−^ DCs, but the mechanistic basis for this alteration in immunogenic properties of TM^+^ DCs is unknown.

We hypothesized that that TM induces tolerogenic DCs by reducing expression of pro-inflammatory molecules in TM^+^ DCs compared to TM^−^ DCs. To test this hypothesis, we investigated the differential expression of genes and miRNA between TM^+^ and TM^−^ dendritic cell sub-populations, followed up with analysis of changes in protein phosphorylation and finally validated the changes by investigating predicted activities.

## Materials and Methods

### Materials

Soluble recombinant human TM (ART123; sTM) consisting of the extracellular domains only was supplied by Asahi Kasei Corporation (Tokyo, Japan). The sTM was clinical grade material approved for use in Japan and does not contain LPS. RPMI 1640 medium was from Sigma (St Louis, MO). Fetal bovine serum (FBS) was from BioWhittaker (Walkersville, MD).

### Mice

Mice used in these experiments were 10 - 12 weeks old Balb/c mice that weighed 17–18 g from Nihon SLC (Hamamatsu, Japan) and housed in the animal facility of Mie University. Mice were maintained on a constant 12-hour light/12-hour dark cycle in a temperature- and humidity-controlled room and were given *ad libitum* access to food and water.

### Ethics Statement

The Mie University Committee on animal investigation approved the experimental protocols, and the experiments were performed according to the guidelines for animal experiments of the National Institute of Health.

### Preparation of DCs

Mouse bone marrow cells from 10 - 12 week old Balb/c mice were grown in RPMI 1640 with 10% FBS and 100 ng/ml granulocyte-macrophage colony-stimulating factor for 6 days as previously described [11], [12]. Some cultures were treated with 200 nM sTM from day 4 to day 6. On day 6, the cultures were separated using rat anti-TM mAb (R&D, Minneapolis, MN) and anti-rat-IgG magnetic microbeads (Miltenyi Biotec, Bergisch Gladbach, Germany) into TM^+^ and TM^−^ DCs. The purity of both the TM^+^ and TM^−^ DC preparations was >93%. Thus this procedure resulted in 4 types of cells: TM-treated TM^+^ DCs, TM-treated TM^−^ DCs, untreated TM^+^ DCs and untreated TM^−^ DCs. In some experiments cell culture supernatants were analyzed by ELISA to determine the levels of lipoxins (Cayman Chemical, Ann Arbor, MI) following the manufacturer’s instructions. Each experiment was carried out on at least two independent occasions and the tests on each culture were performed at least in duplicate.

### Flow Cytometry Analysis

Mouse cells were incubated with anti-CD16/32 for 30 minutes before staining with antibodies against B220, CD4, CD8α, CD11b, CD11c, CD80, CD86, CD205, MHC II and TM or their isotype controls. FACScan (BD Biosciences, San Jose, CA) was used for data acquisition and CellQuest (BD Biosciences) for data analysis.

### Gene Expression Analysis

RNA was prepared from three independent experiments in which DCs were sorted into subpopulations of TM^+^ DCs and TM^−^ DCs whether the cultures had been treated with sTM or not. RNA was extracted from the cells using RNeasy (Qiagen, Valencia, CA) and the amount of RNA was evaluated by NanoDrop ND8000 (Thermo Scientific, Wilmington, DE) while quality was determined by analysis on the Agilent Bioanalyzer 2100 (Agilent Technologies, Santa Clara, CA). The RNA was converted to cDNA and mRNA levels were assayed on Affymetrix GeneChip Mouse Gene 1.0 ST microarrays following the manufacturer’s instructions (Affymetrix, Santa Clara, CA). Levels of specific miRNAs were estimated by hybridization to Affymetrix GeneChip miRNA 2.0 microarrays.

The mRNA data were normalized and summarized for each transcript using the multi-array average (RMA) procedure as implemented in APT software version 1.12.0 and NetAffx Release 31 annotations (Affymetrix, Santa Clara, CA). Further analysis was done using a linear mixed effects model (Methods S1). The microarray data has been deposited in NCBI Gene Expression Omnibus (http://www.ncbi.nlm.nih.gov/geo) with accession number GSE45652.

The raw data from miRNA experiments was imported into Partek Genomics Suite using the robust RMA routine for normalization and summarization (Partek, St. Louis, MO). miRNAs that changed significantly were identified by ANOVA as implemented within Partek Genomics Suite.

### Validation of changes in RNA Levels

Levels of selected mRNAs were analyzed by qPCR using predesigned and validated primer sets (Qiagen). The values were normalized to the expression of *GAPDH*. The data was expressed as mean ± S.D. of relative gene expression to *GAPDH* using the ΔΔCt method.

### Pathway Analysis

Statistical analysis was performed in R [13]. The Benjamini-Hochberg method was used to calculate the false discovery rate (FDR) [14]. Gene set analysis was accomplished using CERNO (Coincident Extreme Ranks in Numerical Observations) [15], which tested each set’s genes for extreme ranks of differential expression among all measured genes.

For the sorted effect of TM^+^ DCs versus TM^−^ DCs, gene sets from Reactome (http://reactome.org/, 2012-02-16 download of human, NCBI Homologene Build 65 and Entrez Gene history from 2012-03-05 for orthology to mouse) with a FDR-corrected CERNO p-value below 10^−4^ and genes in those sets with a transcript PWF quantile below 0.01 or 0.001 were selected (Methods S1). These lists were then used to construct a matrix with gene sets plotted against genes. The order of the rows and columns in the plot were determined by unsupervised clustering according to Canberra distance as implemented in R.

### Phosphoprotein Analysis

Cell lysates of DCs treated with sTM and sorted into TM^+^ and TM^−^ DCs as described above were prepared by washing the cells with ice-cold phosphate-buffered saline before resuspension in lysis buffer (20 mM 3-(N-morpholino)propanesulfonic acid, 2 mM ethylene glycol tetraacetic acid, 5 mM ethylenediamine tetraacetic acid, 30 mM sodium fluoride, 40 mM β-glycerophosphate, 10 mM sodium pyrophosphate, 2 mM sodium orthovanadate, 1 mM phenylmethanesulfonyl fluoride, 3 mM benzamidine, 5 µM pepstatin, 10 µM leupeptin, and 0.5% Triton X-100; final pH 7.0). After sonication for 15 min, the samples were centrifuged at 100,000 rpm for 30 min at 4°C to separate cell debris and the total protein concentration was measured by a dye-binding assay (BCA protein assay kit, Pierce, Rockford, IL) following the manufacturer’s instructions. Phosphoprotein screening was performed in the Kinetworks™ phospho-site broad coverage pathway screen (Catalog Number - KPSS 1.3) by Kinexus (Vancouver, BC, Canada) using a panel of 40 antibodies (Table S2). The resulting blots were quantitated by scanning and proteins that showed a significant change (>20%) were identified.

### Cell Cycle Analysis

Cells were fixed in cold ethanol and washed twice in PBS. After treatment with 20 µg/ml RNase A at 37°C for 30 min, cells were stained with 20 µg/ml propidium iodide and analyzed by FACScan flow cytometer. The percentage of cells in various phases of the cell cycle was calculated.

### Determination of Effect of Neutralizing IL-10

BMDCs were isolated and cultured as described above. On day 4 cultures were treated with either 10 µg/ml irrelevant control rat IgG1κ (BD Bioscience) or 10 µg/ml rat anti-mouse IL-10 antibody clone JES5-2A5 (eBioscience). Some cultures were also treated with 200 nM sTM on day 4. On day 6 the DCs were stained with anti-CD11c and anti-TM antibodies before analysis by flow cytometry. After gating on CD11c^+^ DCs, expression of TM expression was measured.

### Measurement of TM Cofactor Activity on BMDCs

BMDCs were isolated and cultured as described above. The cells were washed three times in reaction buffer (50 mM Tris-HCl, pH 7.5, 150 mM NaCl, 2 mM CaCl_2_, 0.1% bovine serum albumin) and then 10^6^ cells were incubated for 1 h at room temperature in the presence of 100 nM PC or 500 nM proCPB2 with 10 nM thrombin (Mitsubishi Tanabe Pharma Corporation, Osaka, Japan) in a final volume of 80 µL reaction buffer at 37°C under an atmosphere of 95% air and 5% CO_2_. Thrombin was then inhibited by incubating with hirudin (250 antithrombin units) for 10 min. The plates were centrifuged and the generation of aPC and CPB2 determined in the supernatants. PC activation was assessed by measurement of the cleavage of the APC substrate, S2366 (Chromogenix AB, Mölndal, Sweden), using an ELISA microplate reader at 405 nm. CPB2 generation was assessed using the TAFI Activity kit (American Diagnostica, Stamford, CT). The OD values were converted to molar values by comparison to standard curves of aPC and CPB2 generated from aPC that had been purified as PC and activated as described [16] while proCPB2 was purchased from Meridian Life Science, Memphis, TN and activated as described [17].

### Statistics

The statistical analysis of the microarray data and pathways is described above. All other data are expressed as the mean ± standard deviation (s.d.) unless otherwise specified. Statistical analyses were carried out using GraphPad Prism 5.1 (La Jolla, CA) for the Macintosh. When more than two groups were compared, ANOVA was used followed by post hoc analysis with Bonferroni’s multiple comparison test. P<0.05 was considered as statistically significant.

## Results

### Characterization of TM^+^ DCs

**Figure 1:**
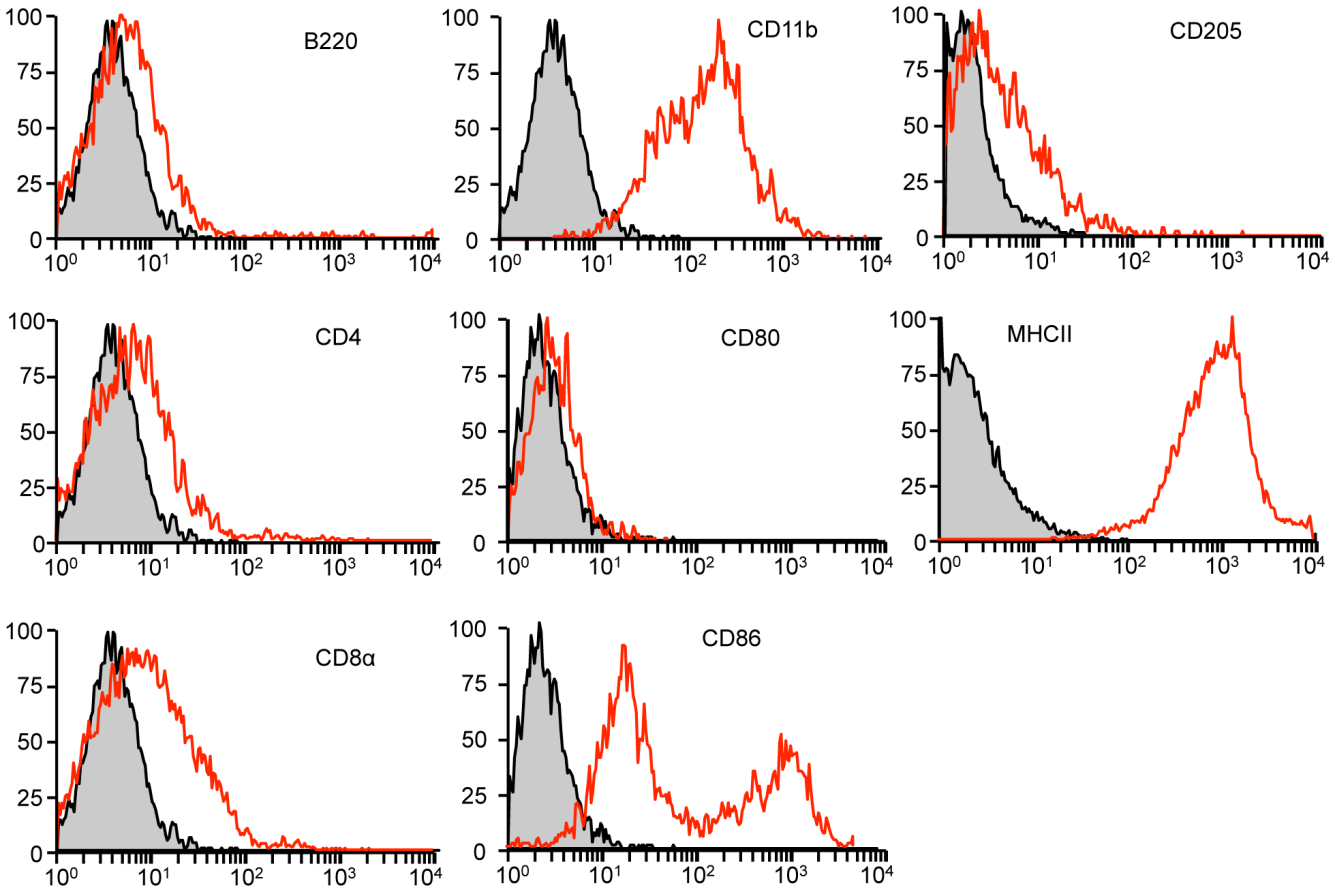
Characterization of TM^+^ DCs. Mouse bone marrow cells were differentiated in GM-CSF for 6 days in the presence of 200 nM sTM from day 4 to 6. DCs were then analyzed for the expression of cell surface markers by flow cytometry cells gated on CD11c^+^ and TM^+^ for the expression of other DC markers. Isotype control is shown in gray.

In order to extend the characterization of TM^+^ DCs derived in vitro from bone marrow cells treated with sTM shown in Table 1 of reference [11], we carried out a flow cytometry analysis of these cells gated on CD11c^+^ and TM^+^. These cells have low expression of B220 (plasmacytoid DC marker), CD4, CD8α, CD80 (DC maturation marker) or CD205 (regulatory DC marker) (Figure 1). In contrast they express high levels of CD11b (conventional DC marker) and MHC II (DC maturation marker) and medium levels of CD86 (DC maturation marker). A similar marker profile is found on DCs isolated from spleen (data not shown), reflecting the presence of this sub-population of DCs *in vivo*. Previously we had shown that in comparison with TM^−^ DCs, expression of DC maturation markers such as CD80, CD86 and MHC II is reduced in TM^+^ DCs which is confirmed here [11]. This data show that the TM^+^ DCs are a distinct sub-population of mouse conventional DCs.

**Table 1:** Validation of microarray data by qPCR and protein levels.

	Microarray	qPCR	FACS/ELISA
CCR5	2.17	1.85	↓
CCR7	−2.68	−2.70	↓
CD206 (MRC1)	6.92	10.01	↓
CD80	−1.80	−1.05	↓
CD83	−4.63	−11.63	↓
CD86	−1.28	ND	↓
CXCR4	−1.24	−2.7	↓
IL-10	−1.36	−210	↑
IL-12p70	A: −6.79; B; −7.02	A: −11.1; B: −39	↓
IL-6	−6.1	−21.9	↓
MHC II H2-A	H2-Aa: 1.29; H2-Ab1∶1.39	6.03	↓
MHC II H2-E	H2-Eb1∶1.31; H2-Eb2∶1.09	15.23	↓
TM (THBD)	1.17	3.06	↑
TNFα	−1.38	2.29	↓

Mouse bone marrow cells were differentiated in GM-CSF for 6 days in the presence of 200 nM sTM from day 4 to 6. DCs were then analyzed for the expression of cell surface markers by flow cytometry while RNA was analyzed on microarrays and by qPCR. In some experiments protein levels in the medium was determined by ELISA. The direction of change in levels of protein is represented by arrows. CD206 is also known as mannose receptor C type 1 (MRC1). The mRNA data shows the results for both the A and B chains of IL-12p70. ND: not determined; the value of the change in gene expression in qPCR was determined by ratio of ΔΔct. The FACS and ELISA data were taken from Takagi et al [11].

### TM^+^ DCs have Lower Expression of Maturation Markers than TM^−^ DCs Irrespective of sTM Treatment

**Figure 2:**
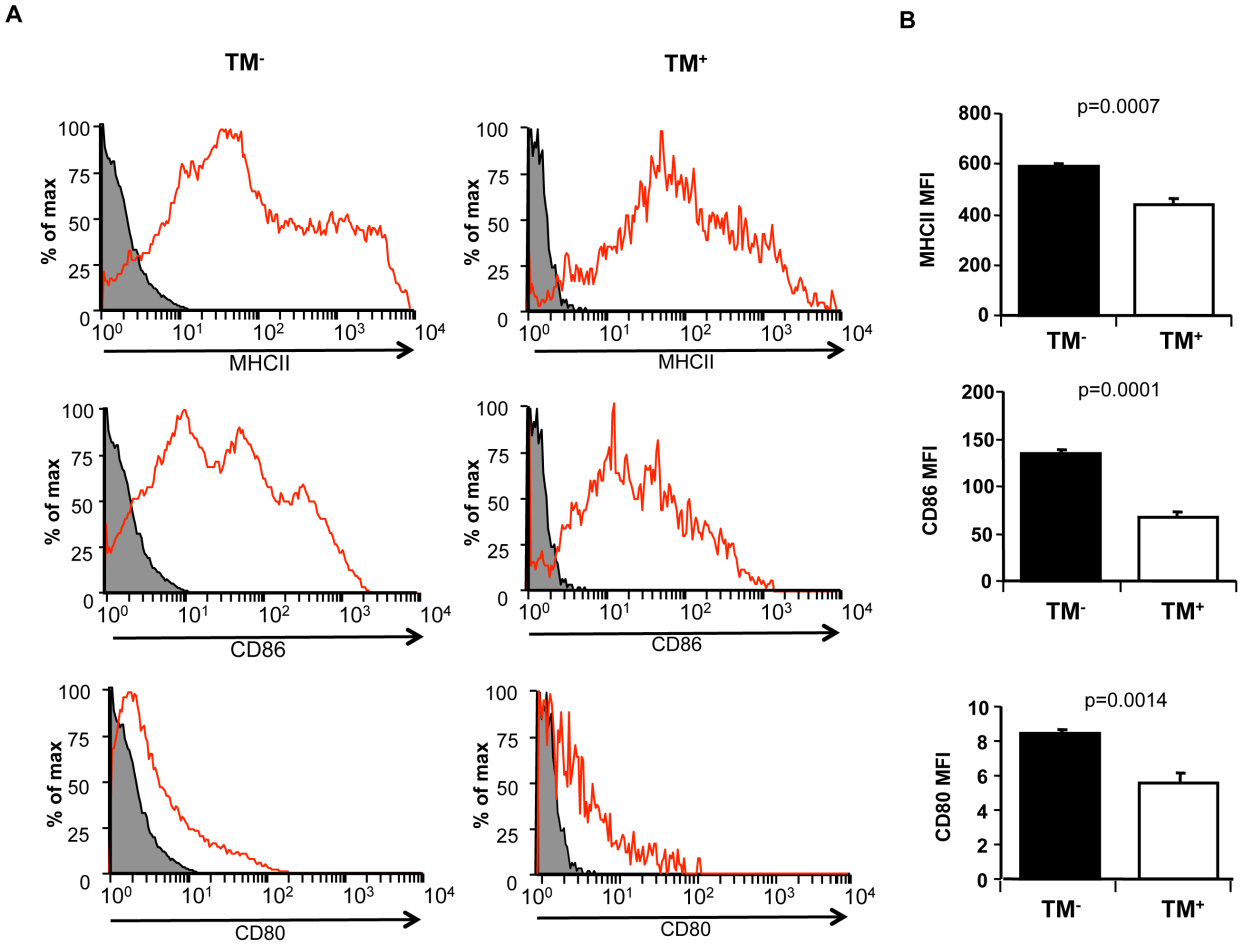
TM^+^ DCs from control cultures have lower expression of maturation markers than TM^−^ DCs. Mouse DC cultures were sorted into TM^+^ and TM^−^ DCs before analysis of MHC II, CD80 and CD86 expression by flow cytometry. (A) Representative experiment is shown with isotype control in gray. (B) The mean fluorescent intensity (MFI) from three independent experiments is shown. Error bars indicate s.d.

Previously we had shown that TM^+^ DCs isolated from mouse bone marrow cell culture following treatment with sTM had reduced expression of maturation markers such as MHC II, CD80, CD83 and CD86 while TM expression was increased from ∼5% to ∼15% [11]. However the TM^+^ DCs present in untreated cultures had not been compared to TM^−^ DCs from the same cultures. Therefore we determined if TM^+^ DCs in untreated cultures also had reduced expression of maturation markers by isolating TM^+^ DCs from untreated cultures and analyzing their expression of maturation markers by flow cytometry. Similarly to TM^+^ DCs from sTM-treated cultures, these TM^+^ DCs from control cultures had reduced levels of expression of MHC II, CD80 and CD86 compared to TM^−^ DCs (Figure 2). Thus, TM^+^ DCs had reduced levels of expression of maturation markers irrespective of whether they had been additionally treated with sTM or not, suggesting that they shared properties in common.

### Differential Gene Expression between TM^+^ and TM^−^ DCs

Based on our earlier data showing that TM^+^ were tolerogenic while TM^−^ DCs were immunogenic [11], we hypothesized that there would be a global change in gene expression when these two cell types were compared, possibly accounting for the change in phenotype. Therefore we compared the levels of specific mRNAs in TM^+^ and TM^−^ DCs by microarray analysis as well as investigated if there were differences in gene expression due to isolating TM^+^ DCs from untreated or sTM-treated cultures.

The microarray data was analyzed by a linear mixed effects model to ensure that probabilities were correctly assessed (Methods S1). We used a model for changes in gene expression in which three covariate terms were included: sorting of DCs into TM^+^ and TM^−^ DCs (sorted); whether the culture had been treated with sTM (treatment) and a term for interaction of the other two terms (interaction). Because ranking by raw F-values does not emphasize transcripts that are specifically dependent on a single factor, we calculated proportion-weighted F (PWF). This ensured that transcripts that showed a strong dependence on a covariate term (e.g., ‘sorted’) were top-ranked for that covariate term, but also de-emphasized it when the model revealed yet stronger effects for other covariate terms for the transcript.

**Figure 3:**
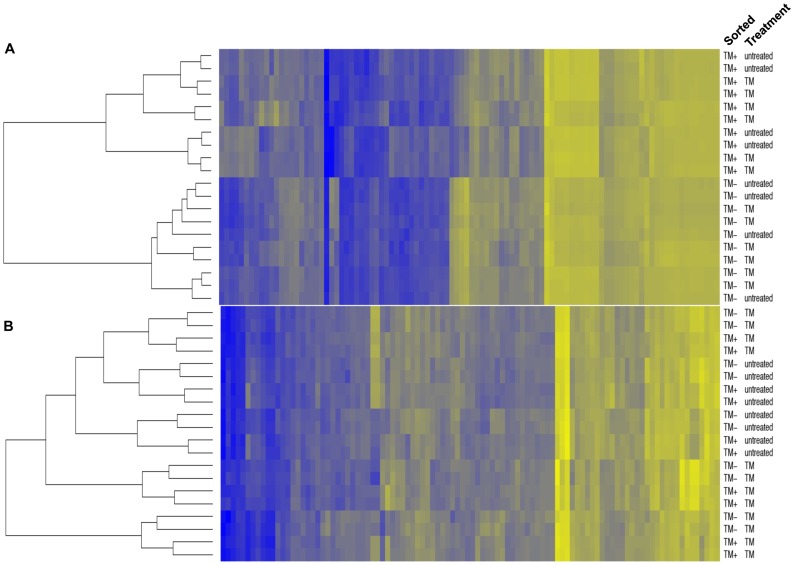
Gene expression is significantly altered in TM^+^ DCs compared to TM^−^ DCs. (A) A heat map was constructed of the top 100 genes that were changed between the TM^+^ and TM^−^ DCs identified as described in Materials and Methods. The list of genes is shown in Table S1a. (B) A heat map of the top 100 genes that were changed between the sTM treated and untreated DCs. Down-regulated genes are in blue; up-regulated genes are in yellow. The depth of the colors is based on rescaled Z-values, with high values being yellow and low values blue. The list of genes is shown in Table S1b.

Gene lists were created for each of the three covariate terms ordered by the value of PWF. Next, these gene lists were used to construct heat maps of the top 100 genes identified for the “sorted” and “treatment” covariates (Fig. 3; Table S1). The heat map of the “sorted” covariate term showed that there were systemic changes with a high degree of significance between gene expression in TM^+^ DCs and TM^−^ DCs while sTM treated DCs had different gene expression than untreated DCs. The unsupervised clustering of the samples clearly segregated TM^+^ DCs from TM^−^ DCs. In contrast, the sTM-treated sorted TM^+^ DCs and sorted TM^−^ DCs were not clustered completely separately. Samples from the sTM-treated cultures tended to cluster together as did those from control untreated samples. The experimental replicates did cluster together which motivated the use of experiment as a random effect in the models. These data suggest that gene expression in TM^+^ DCs is profoundly altered from that observed in TM^−^ DCs and that sTM treatment alters gene expression in DCs independently of the concomitant increase in TM^+^ DCs.

**Figure 4:**
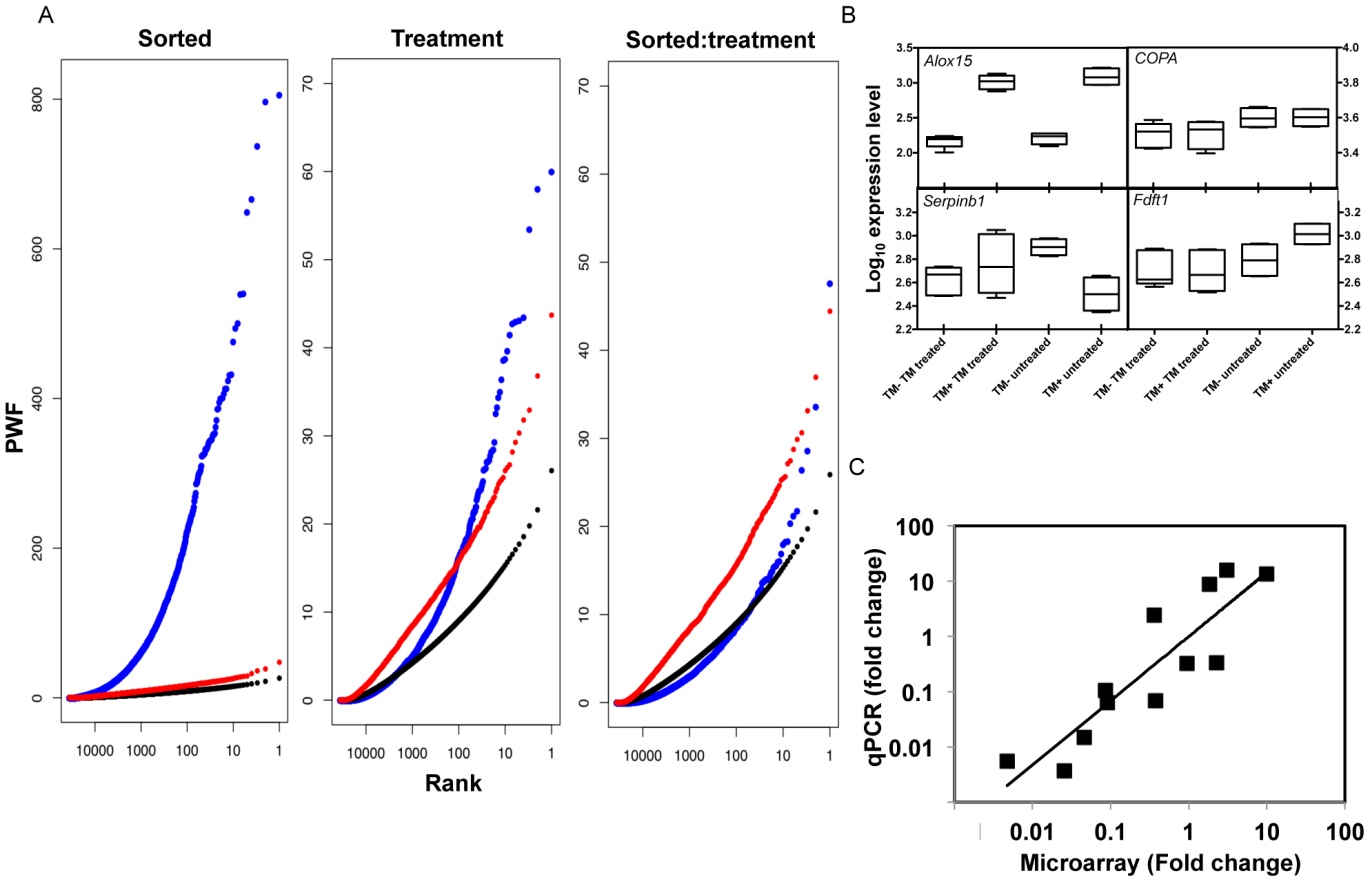
Gene expression changes in DCs following sTM treatment or sorting into TM^+^ or TM^−^ DCs. (A) The mean (black) and 95th percentile (red) of PWF values for each probe rank from 500 permutations of the sample-labels as described in Materials and Methods show that the ‘sorted’ term PWF values (blue) were extraordinarily large compared to the simulations. For the ‘treatment’ term, the PWF values of fewer than 1000 probes were well above the simulated values, and for the ‘sorted:treatment’ interaction term, few probes showed a significantly higher PWF than by permuted sample-label computations. (B) PWF statistics permitted identification of genes exhibiting the strongest association with sorting into TM^+^ and TM^−^ DC, for example *Alox15*. Similarly, genes exhibiting strong association with TM treatment included *Copa*. *Serpinb8* exhibited a large PWF value for the sorted:treatment interaction, as seen by the contrasting effect of treatment on gene expression in TM^−^ and TM^+^ DCs. *Fdft1* gave large values of PWF for both treatment and sorted:treatment interaction, and shows both down-regulation with TM treatment and notably higher expression in TM^+^ DCs, specifically in untreated cells. (C) Correlation between gene expression changes determined by microarray to those determined by qPCR. A panel of genes (Table 1) whose expression was changed when analyzed by microarray, their fold change was also determined by qPCR. The qPCR data was normalized to GAPDH resulting in this equation for the best fit line: log y = (0.99 *log x) +1.16 with R^2^ = 0.79.

In order to assess the significance of these changes the PWF for individual transcripts was compared to a simulated data set. TM^+^ and TM^−^ DCs exhibited quite distinct expression patterns for a large number of genes for thousands of transcripts, with the sorted term well above values found by randomly permuted sample-label computations in simulations (Fig. 4A). Comparing sTM-treated DCs to untreated DCs did not result in such a distinct contrast in gene expression, as shown by the difference in scale of the Y axis. Nevertheless the expression of many genes was strongly associated with treatment with sTM. In contrast we found no evidence that the sorted:treatment interaction term provided very surprising PWF values, compared to the simulation, although top-ranking probes do exhibit patterns consistent with an interaction. Thus, the comparison of the actual data to the simulated data sets generated by random permutation of the sample-labels demonstrates that the strongest differences in gene expression are in the “sorted” covariate, then less strong in the “treatment” covariate and least in the interaction between the two, “sorted:treatment”. Based on the strength of the differences, in the remainder of the studies, we focused on the gene expression differences between TM^+^ and TM^−^ DCs.

### Changes in Genes Representative of Sorting, Treatment and Interaction

To illustrate the representative expression pattern of genes that ranked favorably for terms in the mixed effects model, expression of four genes is presented in Fig. 4B. Using PWF statistics, we identified genes exhibiting the strongest association with sorting into TM^+^ and TM^−^ DC (“sorted”), for example arachidonate 15-lipoxygenase (*Alox15*). In this case, expression of *Alox15* is increased in TM^+^ DCs compared to TM^−^ DCs irrespective of whether or not they had been treated with sTM. Similarly, genes exhibiting strong association with sTM treatment included non-clathrin-coated vesicular coat protein-alpha (*Copa*) in which it can be seen that treatment with sTM reduced expression of *Copa* irrespective of whether the DCs were TM^+^ or not. Serpin peptidase inhibitor B8 (*Serpinb8*) exhibited a large PWF value for the “sorted:treatment” interaction, as seen by the contrasting effect of treatment on gene expression in TM^−^ and TM^+^ DCs. Farnesyl-diphosphate farnesyltransferase (*Fdft1*) ranked favorably for both “treatment” and “sorted:treatment” interaction, and showed both down-regulation with TM treatment and notably higher expression in TM^+^ DCs, specifically in untreated cells.

### Validation of Microarray Data

To validate the data from the microarrays, we determined changes in the levels of mRNA by qPCR for a panel of 14 genes. In all cases except TNF-α, the direction of change of mRNA levels was the same, irrespective of whether the level of mRNA was determined by microarray or by qPCR (Table 1). When fold change determined by microarray analysis was plotted against fold change measured by qPCR, an excellent correlation (p<0.0001) was found with R^2^ = 0.79 (Fig. 4C). There was 2.76 fold more change when gene expression was compared by qPCR than by microarray if β-actin (*ATCB*) rather than *Gapdh* is used as a housekeeping gene to normalize the data (data not shown).

In our previous paper [11], changes in levels of several proteins had been quantitated either when TM^+^ and TM^−^ DCs were compared by flow cytometry or when conditioned media was evaluated by ELISA from cultures of TM^+^ or TM^−^ DCs. We used these data to compare if the direction of changes in gene expression found on the microarrays was the same as that of the proteins and found that there was very good concordance (Table I). Out of 13 proteins whose expression levels had been measured in TM^+^ compared to TM^−^ DCs, only three were not in concordance (CD206, IL10 and MHC II). MHC II protein expression has been shown to be controlled by several mechanisms such as invariant chain degradation and re-endocytosis, maybe explaining the lack of concordance [18], [19]. When the fold change in protein levels were correlated with the fold change in mRNA determined by microarray, a correlation was found (R^2^ = 0.23) but it was much weaker than the comparison of mRNA levels measured by the two techniques. It is expected that the correlation between changes in protein levels and mRNA is lower than that between mRNA determined by different techniques because there are many other parameters apart from mRNA levels that affect protein levels such as protein turnover and control of mRNA translation.

### Alterations in miRNA Expression in TM^+^ DCs Compared to TM^−^ DCs

Previous reports had shown the involvement of miRNAs, such as miR146a [20] and miR155 [21] in determining DC properties [22]. Based on this, we investigated if expression of any miRNA was altered when TM^+^ DCs were compared to TM^−^ DCs. Levels of miRNA were determined by hybridization on an array that displays the full complement of miRNAs present in the genome. miRNAs with significant changes in expression were identified (Table 2) including miRNA146, and miRNA155 whose levels were lower in TM^+^ DCs and miRNA27a* whose level was increased. The changes in levels of miRNA146, and miRNA155 were validated by qPCR confirming the microarray data (data not shown).

**Table 2:** List of miRNAs modulated in TM^+^ DCs compared to TM^−^ DCs.

miRNA	p-value	Fold-Change
miR-146a	1.15E-09	−4.82
miR-31	9.66E-08	−4.95
miR-155	1.97E-07	−3.82
miR-2134	2.13E-07	−2.05
miR-711	1.63E-06	−4.10
miR-3473	1.95E-06	−5.62
miR-574-3p	3.32E-06	−2.55
miR-1195	6.59E-06	2.26
miR-27a*	6.89E-06	14.92
miR-27b*	7.35E-06	2.92
miR-34c	1.80E-05	−3.43
miR-1931	3.34E-05	−2.30
miR-874	4.07E-05	−2.01
miR-196b	7.99E-05	2.59
miR-181a-1*	8.02E-05	4.15
miR-187	8.11E-05	2.02

List of miRs whose expression is altered, identified from microarray analysis by comparison of levels in TM^+^ and TM^−^ DCs which change >2 fold with p<0.0001.

### Difference in Cell Cycle and Inflammatory Genes

**Figure 5:**
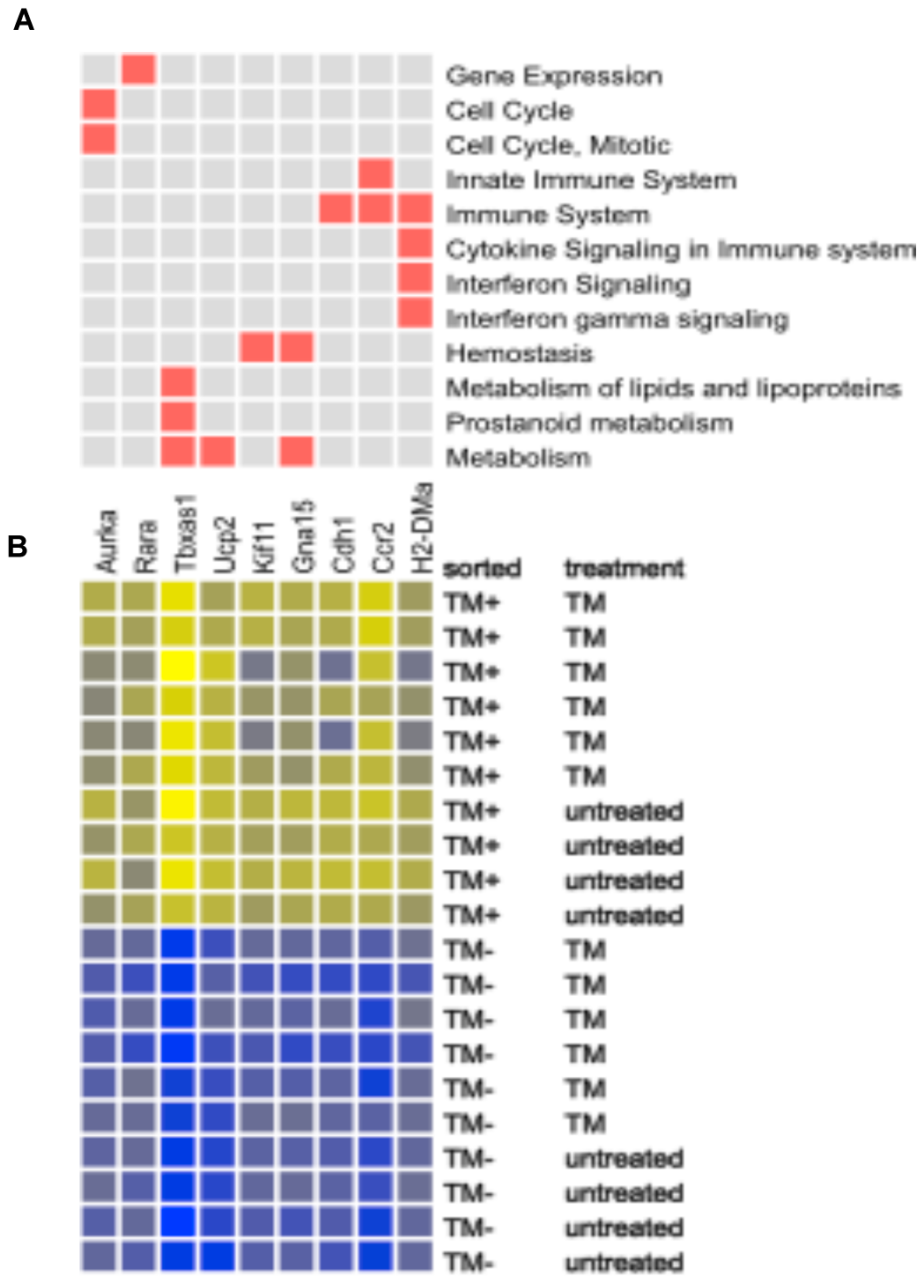
Cell cycle and inflammatory pathways are modulated in TM^+^ DCs compared to TM^−^ DCs. (A) The 12 gene sets identified as described in Materials and Methods are plotted against the top 9 genes (restricted to PWF quantile <0.001) within those sets that changed significantly. A fuller set is displayed in Figure S1. A square is colored if a gene (column) belongs to a set (row). (B) Heat map of the genes identified by the CERNO analysis of gene sets. Yellow is up-regulated, and blue is down-regulated. The depth of the colors is based on rescaled Z-values, with high values being yellow and low values blue.

In order to extend the analysis beyond the level of individual genes, a pathway analysis was carried out to identify those pathways (gene sets) that were significantly altered when TM^+^ DCs are compared to TM^−^ DCs. Fig. 5 plots a design structure matrix with 12 gene sets and 9 genes using stringent thresholds for both gene sets and genes. Figure S1 shows the overlapping 100 gene sets and 77 genes when using a less stringent gene threshold. In both cases, in the top plot a square is colored if a gene (column) belongs to a set (row) while the bottom plot shows a heat map based on the genes in these pathways showing the normalized expression value for each sample.

Several of the gene sets (pathways) identified are involved with the cell cycle such as “Cyclin A/B1 associated events during G2/M transition”, “Cell cycle”, and “G2/M checkpoints,” suggesting that there is a difference in cell division between TM^+^ and TM^−^ DCs. A second prominent cluster of gene sets is involved with inflammation such as “Cytokine Signaling in Immune system” and “Innate Immune System” consistent with previously observed differences in the inflammatory responses of TM^+^ and TM^−^ DCs [11].

### Changes in Expression of Genes in the Hemostasis Set

As TM is a protein that, amongst other properties, can modulate coagulation, it is of interest that expression of the gene set “Hemostasis” was identified as being altered. Within the coagulation cascade genes whose expression was altered include tissue factor (*F3*) that initiates coagulation in the extrinsic pathway was down-regulated in TM^+^ DCs, while prekallikrein (*Pk*) which is involved with the contact phase in the intrinsic pathway and coagulation factor V (*F5*) in the common pathway were both up-regulated in TM^+^ DCs (Table 3). Of the protease activated receptors (PARs) that can serve as thrombin receptors, only the expression of PAR-3 (*F2rl2*) was altered, being up-regulated in TM^+^ DCs. Endothelial Protein C Receptor (EPCR; *Procr*) was down-regulated in TM^+^ DCs as was one of the sphingosine-1-phosphate receptors (*S1pr3*) that are potential downstream genes from EPCR [23]. In contrast, expression of another S1PR gene, *S1pr1*, also downsteam from EPCR was up-regulated in TM^+^ DCs.

**Table 3:** Altered expression of hemostasis genes’, EPCR and Sphingosine phosphate receptors’ mRNA.

	TM^+^ DCs/TM^−^ DCs
EPCR	−3.5
S1PR1	1.9
S1PR3	−3.7
F5	3.46
PAI-1	−5.66
Tissue Factor	−5.83

Change in levels of expression of mRNA encoding some genes involved with hemostasis and down stream effects on inflammation, identified from the pathway analysis as part of the “Hemostasis” set.

### Difference in Arachidonic Acid Metabolism Pathways

One of the genes identified as having significantly changed expression in this analysis is lipoxygenase 15 (*Alox15*; 15LO) which is part of several inflammatory gene sets such as “TRAIL signaling pathway”, “Class 1 PI3K signaling events” and “EGF receptor (ErbB1) signaling pathway”. Several other enzymes involved with arachidonic acid metabolism are modestly increased including lipoxygenase 5 (*Alox5*; 5LO) while lipoxygenase 12 (*Alox12*; 12LO), both genes encoding prostaglandin E synthase 2 (*Ptges2* and *Ptges3*) and cyclooxygenase-2 (Ptgs2; COX-2) are significantly reduced. These changes will be expected to reduce pro-inflammatory prostaglandins synthesis and increase production of the anti-inflammatory lipoxins [24], [25].

### Changes in Protein Phosphorylation

**Figure 6:**
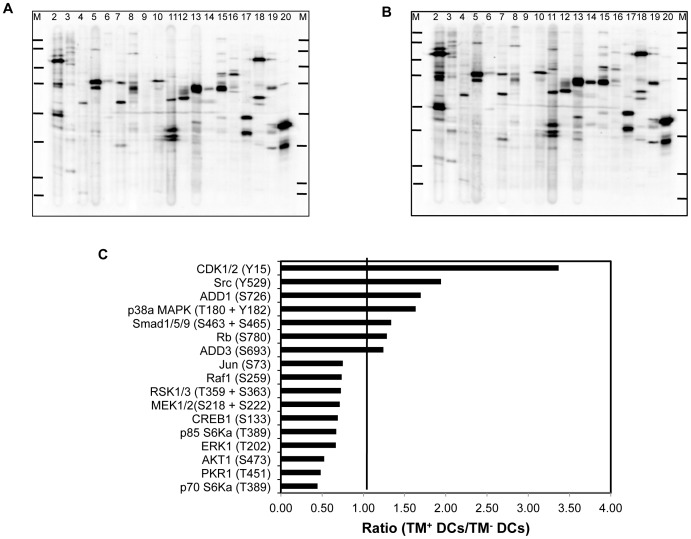
Alterations in phosphorylation of proteins in TM^+^ compared to TM^−^ DCs. Cell lysates were analyzed by Western blots in which individual tracks were probed with a panel of specific anti-phosphoprotein antibodies, developed and scanned. The values from TM^+^ DCs were compared to those from TM^−^ DCs as a ratio. The antibodies used are described in Table S2. The lines in lanes 1 and 21 represent the migration of the marker proteins. (A) representative Western blot of phosphoproteins from TM^+^ DCs. (B) representative Western blot of phosphoproteins from TM^+^ DCs. (C) Quantitation of Western blots showing phosphoproteins whose level had changed by >25%.

In order to investigate if signaling pathways were altered in TM^+^ DCs when compared to TM^−^ DCs, the phosphorylation status of 40 phosphoproteins was carried out by analysis in Western blots of signals from specific anti-phosphoprotein antibodies (Table S2) that had been selected to represent a variety of cellular processes. These showed that there are significant changes in levels of several phosphoproteins when TM^+^ DCs were compared to TM^−^ DCs (Fig. 6). In particular, some phosphoproteins involved in cell cycle control were up-regulated (CDK1/2, Rb). In some cases such as CDK1/2, the mRNA encoding the protein was also up-regulated in TM^+^ DCs, confirming that an increase occurred both at the mRNA level as well as in its activity.

Interestingly, two adducin genes (*Add1* and *Add3*) have increased levels of phosphorylation in TM^+^ DCs that correlates with increased levels of mRNA encoding all three all forms of adducin (ADD1, ADD2 or ADD3) found in the microarray analysis. Phosphorylation of adducins has been linked to cell motility and cell shape [26], [27]; this change in adducin expression and phosphorylation may explain the different morphology of TM^+^ DCs compared to TM^−^ DCs [11].

There is increased phosphorylation of proteins involved with cytokine signaling such as p38a MAPK and SMAD 1/3/5 in TM^+^ DCs compared to TM^−^ DCs. In contrast, there was down-regulation of phosphorylation of other signaling kinases such as MEK1/2 and ERK1. These data suggest that responses to cytokines will be different in TM^+^ DCs compared to TM^−^ DCs.

### More TM^+^ than TM^−^ DCs are in Cell Cycle

**Figure 7:**
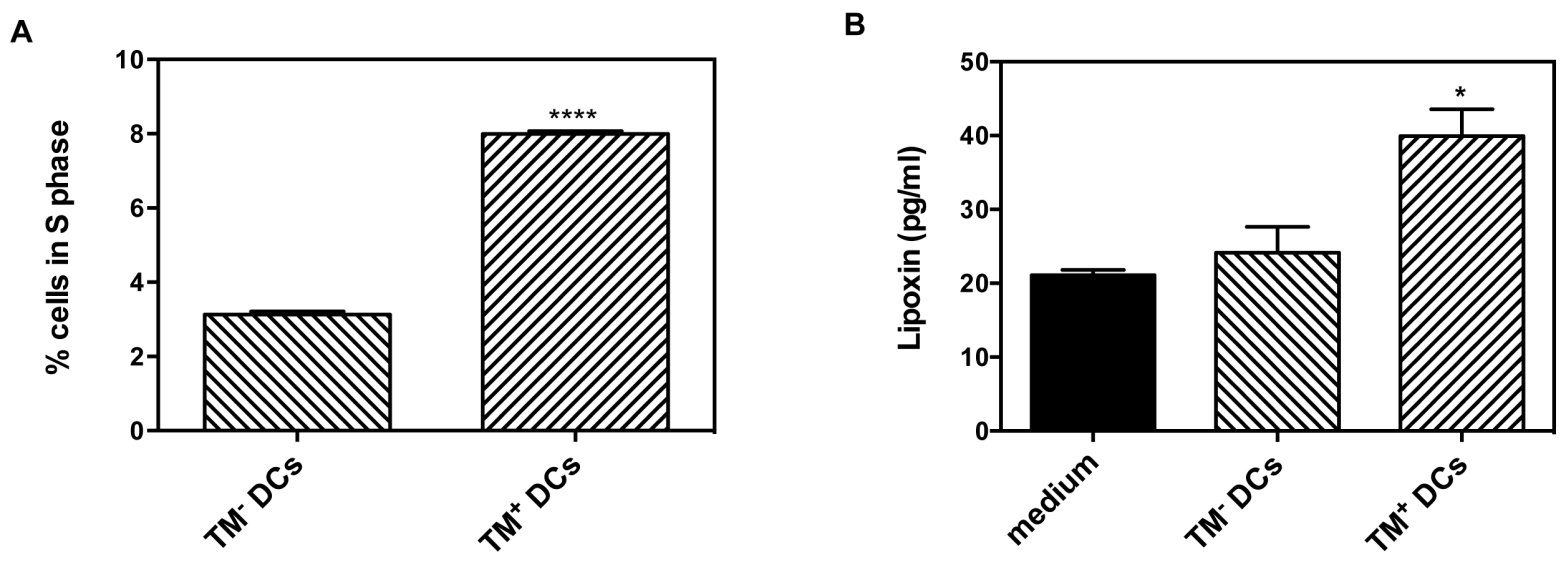
TM^+^ DCs have altered cell cycle, lipoxin production. (A) DCs were cultured in the presence of sTM before sorting into TM^+^ and TM^−^ DCs. Cells were labeled with propidium iodide and analyzed by flow cytometry after 24 hr in culture. The percentage of cells in S phase was calculated. Data was analyzed by Students t test. ****p<0.0001. The mean of 3 experiments is shown with error bars indicating ± sem. (B) DCs were cultured in the presence of sTM before sorting into TM^+^ and TM^−^ DCs. Lipoxin in conditioned medium was determined by ELISA. Data were analyzed by one-way ANOVA followed by post hoc Bonferroni correction. *p<0.05. The mean of 3 experiments is shown with error bars indicating ± sem.

Based on the pathway analysis data, we predicted that more TM^+^ DCs than TM^−^ DCs would be actively in the cell cycle. To test this, we determined the fraction of cells that were dividing by flow cytometry of propidium iodide-labeled cells and demonstrated that there were more TM^+^ DCs in S phase than TM^−^ DCs (TM^+^ DCs: 8.00±0.07% vs. TM^−^ DCs: 3.13±0.08%, p<0.0001; Fig. 7A).

### TM^+^ DCs have Altered Arachidonic Acid Metabolism Compared to TM^−^ DCs

Analysis of the pathway data also predicted that arachidonic acid metabolism would be modified in TM^+^ DCs such that levels of lipoxins would be increased. The levels of lipoxin were measured in cell culture medium and were found to be significantly increased in the medium from TM^+^ DCs compared to levels in medium alone or in medium from TM^−^ DCs (TM^+^ DCs: 40±3.6 pg/ml vs TM^−^ DCs: 24±3.5 pg/ml vs medium: 21±0.7 pg/ml p<0.05 for TM^+^ DCs vs TM^−^ DCs or medium; Fig. 7B). This change in lipid mediators demonstrate that the changes in mRNA encoding key enzymes in arachidonic acid metabolism result in changes in the quantities of their products produced by the DCs and that these changes are consistent with the alteration in properties between TM^+^ and TM^−^ DCs.

### Antagonism of IL-10 Prevents Induction of TM^+^ DCs by sTM

**Figure 8:**
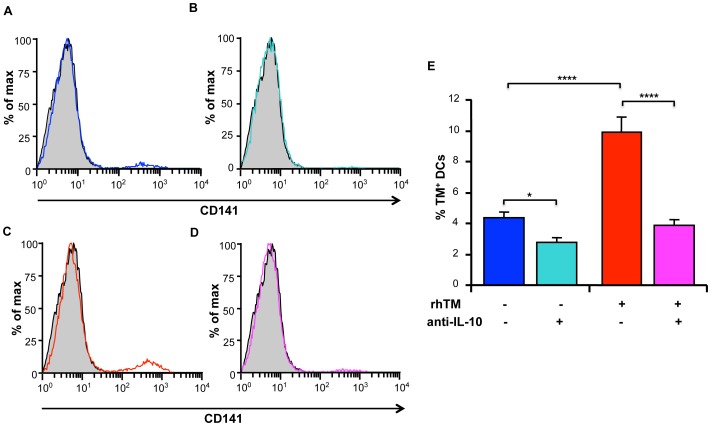
Antagonism of IL-10 prevents induction of TM on DCs treated with sTM. DCs were cultured in the presence of sTM from day 4. Neutralizing anti-mouse IL-10 mAb (clone: 2A5) or irrelevant control IgG were added on day 4 at a final concentration of 10** µ**g/ml. Cells were collected on day 6 and stained with mAb to CD11c and TM. CD11c^+^ cells were gated and expression of TM was analyzed by flow cytometry. Isotype control is shown in gray. (A) Untreated DCs with added control rat IgG. (B) Untreated DCs with added anti-IL-10 mAb. (C) sTM treated DCs with added control IgG. (D) sTM treated DCs with added anti-IL-10 mAb. (E) The mean percentage of TM^+^ DCs was calculated from three experiments is shown with error bars indicating ± sd. Data were analyzed by one-way ANOVA followed by post hoc Bonferroni correction. *p<0.05 and ****p<0.0001.

Because of the importance of IL-10 as an anti-inflammatory moiety as well as its non-concordance between RNA and protein expression, we investigated further its role in inducing TM^+^ DCs. Either untreated DCs or DCs treated with sTM were cultured from day 4 in the presence of a mAb that neutralizes IL-10 before analysis by flow cytometry on day 6 (Fig. 8). In the absence of sTM, the neutralizing antibody significantly reduced the percentage of TM^+^ DCs (untreated+control IgG: 4.36±0.38%; untreated+anti-IL-10 mAb: 2.78±0.38%, p<0.05). Similarly in the presence of sTM the increased percentage of TM^+^ DCs was also reduced by inclusion of the neutralizing anti-IL-10 mAb in the culture (sTM treated+control IgG: 9.94±0.93%; sTM treated+anti-IL-10 mAb: 3.87±0.38%; p<0.0001). These results suggest that IL-10 may be mediating part of sTM’s effects on DCs.

### TM^+^ DCs Activate proCPB2 and Protein C

**Figure 9:**
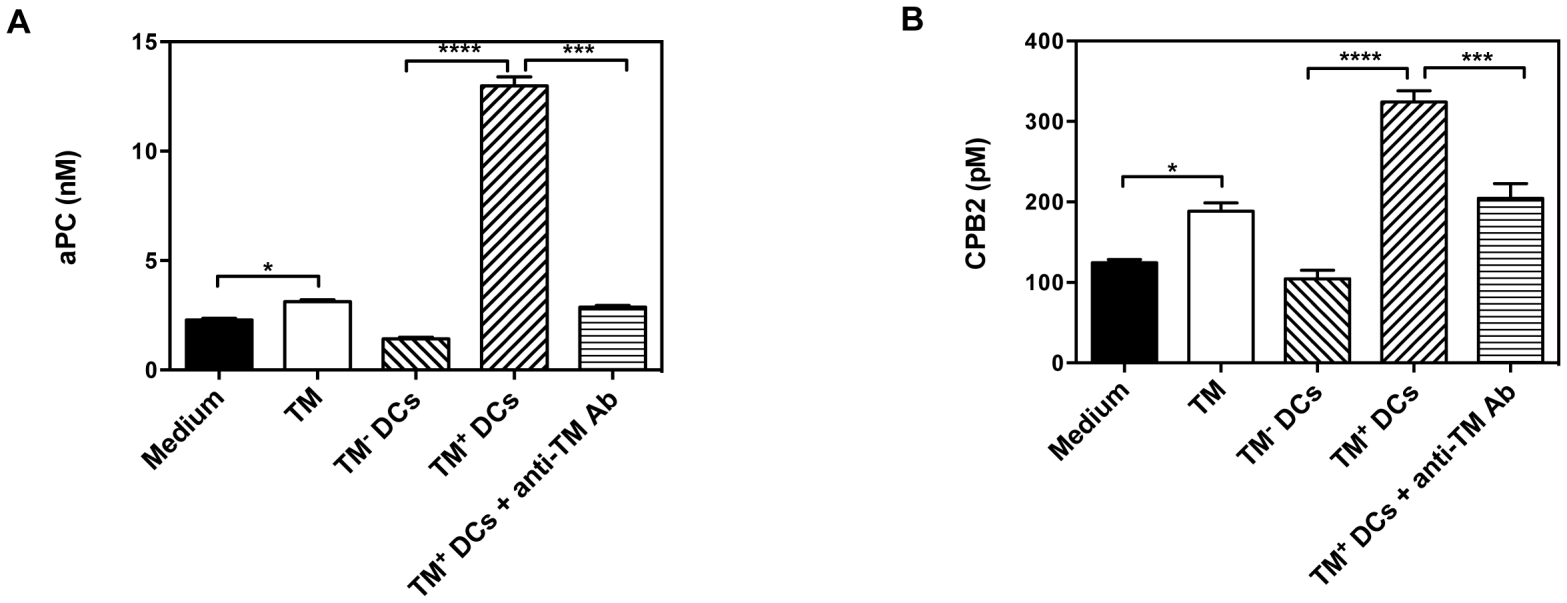
TM^+^ DCs can activate PC and proCPB2. DCs were cultured in the presence of rhTM before sorting into TM^+^ and TM^−^ DCs. (A) Thrombin and PC were incubated with the cells and the generation of aPC was measured. Data were analyzed by one-way ANOVA followed by post hoc Bonferroni correction. *p<0.05; ***p<0.001 and ****p<0.0001. The mean of 3 experiments is shown with error bars indicating ± sem. (B) Thrombin and proCPB2 were incubated with the cells and the generation of CPB2 was measured. Data were analyzed by one-way ANOVA followed by post hoc Bonferroni correction. *p<0.05; ***p<0.001 and ****p<0.0001. The mean of 3 experiments is shown with error bars indicating ± sem.

Previous studies have demonstrated the presence of TM on the cell membrane of DCs but whether it can activate proCPB2 and protein C is unknown. To clarify this we differentiated DCs from mouse bone marrow cells, separated them into TM^+^ and TM^−^ DCs and then cultured them in the presence of thrombin and PC or proCPB2. After inhibiting thrombin, the conditioned medium was then assayed for the presence of either aPC or CPB2. The results showed enhanced generation of aPC and CPB2 in the supernatants of TM^+^ DCs compared to TM^−^ DCs (Fig. 9A & 9B). The enhanced activation of PC and proCPB2 was inhibited by including a polyclonal anti-mouse TM antibody in the incubation medium, demonstrating that the activation of PC and proCPB2 was dependent on the TM expressed on DCs.

## Discussion

Earlier we had shown that adoptive transfer of TM^+^ DCs into naïve mice protected them from airway hypersensitivity while adoptive transfer of TM^−^ DCs exacerbated the disease [11]. Here we show that even in cultures that had not been treated with sTM, the TM^+^ DCs had reduced expression of maturation markers similar to that seen in the cultures that had been treated with sTM. In order to understand in more detail the differences in properties between TM^+^ and TM^−^ DCs as well as to investigate the effect of sTM treatment, in this study we analyzed the DCs for alterations in mRNA and miRNA levels and phosphorylation of proteins. In all of the analyses, major differences were identified between TM^+^ and TM^−^ DCs suggesting that expression of TM on the cell surface was indicative of major changes in gene expression and cell signaling. Levels of mRNA were also modulated by treatment with sTM as well as, in some cases, an interaction between sTM treatment and the status of TM expression on the DCs.

IL-10 is an important anti-inflammatory cytokine whose levels were decreased in TM^+^ DCs compared to TM^−^ DCs in the RNA analyses but increased in the protein analysis. When the role of IL-10 was investigated further by antagonizing it with a neutralizing antibody, the percentage of TM^+^ DCs was decreased. That suggests that IL-10 plays a role in the induction of TM^+^ DCs both in control cultures as well as in ones treated with sTM and implies that IL-10 is downstream from the interaction of sTM with DCs. The data from the treatment with anti-IL-10 mAb is consistent with the presence of IL-10 in the medium. The lack of concordance between IL-10 mRNA and proteins levels could be reconciled as IL-10 protein was determined by ELISA of the culture medium, which represents the accumulated production between day 4 and day 6. In contrast the mRNA levels measure the amount present in the DCs on day 6. If there is an early response to sTM treatment causing transient production of IL-10 then the results of RNA and protein analyses might be discordant. In the human system IL-10 treatment has been shown to induce tolerance in monocyte derived DCs [28] consistent with the data shown here on DCs derived from mouse bone marrow.

As miRNAs can control levels of mRNA and their translation, we determined if any miRNAs were significantly modulated when TM^+^ DCs were compared to TM^−^ DCs. Levels of several miRNAs such as miR-27a*, miR-31, miR-146a and miR-155 were found to be significantly altered. miR-31 down-regulates cell adhesion molecules [29] and miR-27a* reduces the cytotoxicity of NK cells by down-regulating perforin1 and granzyme B expression [30]. Antigen presentation is regulated by miR-155 while miR-146a is involved in innate immunity through regulation of Toll-like receptor signaling and cytokine responses [31], [32]. Thus these miRNAs may be playing similar roles to those reported in modulating the phenotype of TM^+^ DCs.

For an overview of the changes at a different level, the status of phosphorylation on 40 phosphoproteins was analyzed in TM^+^ DCs. Confirming the data from the mRNA analysis, changes were found in kinases that control the cell cycle as well as in cytokine signaling. The changes in phosphoprotein status were concordant with the changes in gene expression. Interestingly, the phosphorylation of adducin-α (*Add1*) and adducin–γ (*Add3*) were increased in TM^+^ DCs as was the mRNA encoding adducin-γ. Adducins cap the actin filaments and phosphorylation disrupts the cytoskeleton [33], which could lead to the changes in cell shape and motility observed in TM^+^ DCs.

When pathway analysis was carried out on the genes whose expression was altered when TM^+^ DCs were compared to TM^−^ DCs, a number of intriguing gene sets was identified. Several of the identified pathways have clear functions in inflammation and the immune system and therefore may contribute to the mechanism by which TM^+^ DCs become tolerogenic. Several genes affecting metabolism of arachidonic acid were modulated, suggesting that alterations in levels of prostenoids or thromboxane might be involved. One of the genes whose expression is altered is dipeptidase2 (*Dpep2*) that is responsible for the conversion of leukotriene D4 to leukotriene E4 that may mediate many of the features of asthma [34], [35]. We compared the levels of lipoxin in culture medium from TM^+^ DCs with those from TM^−^ DCs and found that it was increased. As lipoxins play an anti-inflammatory role as well as promote the resolution of inflammation [36], [37], the increase in lipoxin levels in TM^+^ DCs is consistent with their tolerogenic role recently reported in asthma [38].

Another group of gene sets that was identified by the comparison of TM^+^ to TM^−^ DCs is involved with pathways controlling the cell cycle. Among these gene sets, cyclins and cell division kinases (e.g., CDK1) were increased in TM^+^ DCs suggesting that more of these cells were dividing. The changes in gene sets for ribosome synthesis and protein translation are consistent with an increase in cell division. Measurement of the proportion of dividing cells confirmed this prediction, thereby showing that the analyses of the levels of mRNA, miRs and protein phosphorylation have uncovered new biological properties of the TM^+^ DCs.

Previous studies have shown that TM is expressed on the surface of DCs but its function has not been characterized [11], [39], [40]; in particular, the role of TM on DCs in the generation of aPC and CPB2 was unknown. In the present study, we found that TM on DC surface possesses cofactor activity for activation of protein C and proCPB2 by thrombin. Induction of TM on the DC surface would localize the production of aPC and CPB2 to that surface.

Taken together our data shows that gene expression in TM^+^ DCs is profoundly different from that in TM^−^ DCs leading to changes in observable phenotype such as lipoxin production, rate of cell division and TM activity. These phenotypic alterations are consistent with the change from TM^−^ DCs being immunogenic to TM^+^ DCs being tolerogenic.

## Supporting Information

Figure S1 **The top 100 gene sets identified as described in Materials and Methods are plot as a design structure matrix against the top 77 genes within those sets that changed significantly.** A square is colored if a gene (column) belongs to a set (row). B. Heat map of the genes identified by the CERNO analysis of gene sets. Yellow is up-regulated, and blue is down-regulated. The depth of the colors is based on rescaled Z-values, with high values being yellow and low values blue.
(PDF)

Table S1 **Top 100 probes changed between TM+ and TM- DCs (A); Top 100 probes changed between sTM treated and untreated DCs (B).**
(XLSX)

Table S2 **Antibodies used to detect phosphoproteins in** **Figure 6** **.**
(XLSX)

Methods S1 **Ranking of transcripts using linear mixed effects model statistics.**
(DOCX)

## References

[1] Esmon NL, Owen WG, Esmon CT (1982) Isolation of a membrane-bound cofactor for thrombin-catalyzed activation of protein C. J Biol Chem. 257: 859–864. 6895633

[2] Suzuki K, Kusumoto H, Deyashiki Y, Nishioka J, Maruyama I, et al (1987) Structure and expression of human thrombomodulin, a thrombin receptor on endothelium acting as a cofactor for protein C activation. EMBO J 6: 1891–1897. 2820710 10.1002/j.1460-2075.1987.tb02448.x PMC553573

[3] Morser J (2012) Thrombomodulin links coagulation to inflammation and immunity. Curr Drug Targets 13: 421–431. 22206250 10.2174/138945012799424606

[4] Nesheim M, Wang W, Boffa M, Nagashima M, Morser J, et al (1997) Thrombin, thrombomodulin and TAFI in the molecular link between coagulation and fibrinolysis. Thromb Haemost 78: 386–391. 9198184

[5] Wang W, Nagashima M, Schneider M, Morser J, Nesheim M (2000) Elements of the primary structure of thrombomodulin required for efficient thrombin-activable fibrinolysis inhibitor activation. J Biol Chem 275: 22942–22947. 10801821 10.1074/jbc.M001760200

[6] Morser J, Gabazza EC, Myles T, Leung LL (2010) What has been learnt from the thrombin-activatable fibrinolysis inhibitor-deficient mouse? J Thromb Haemost 8: 868–876. 20128866 10.1111/j.1538-7836.2010.03787.x

[7] Esmon CT (2003) The protein C pathway. Chest 124: 26S–32S. 12970121 10.1378/chest.124.3_suppl.26s

[8] Conway EM, Van de Wouwer M, Pollefeyt S, Jurk K, Van Aken H, et al (2002) The lectin-like domain of thrombomodulin confers protection from neutrophil-mediated tissue damage by suppressing adhesion molecule expression via nuclear factor kappaB and mitogen-activated protein kinase pathways. J Exp Med 196: 565–577. 12208873 10.1084/jem.20020077 PMC2193995

[9] Geudens N, Van de Wouwer M, Vanaudenaerde BM, Vos R, Van De Wauwer C, et al (2008) The lectin-like domain of thrombomodulin protects against ischaemia-reperfusion lung injury. Eur Respir J 32: 862–870. 18508817 10.1183/09031936.00157107

[10] Van de Wouwer M, Plaisance S, De Vriese A, Waelkens E, Collen D, et al (2006) The lectin-like domain of thrombomodulin interferes with complement activation and protects against arthritis. J Thromb Haemost 4: 1813–1824. 16879225 10.1111/j.1538-7836.2006.02033.x

[11] Takagi T, Taguchi O, Toda M, Ruiz DB, Bernabe PG, et al (2011) Inhibition of allergic bronchial asthma by thrombomodulin is mediated by dendritic cells. Am J Respir Crit Care Med 183: 31–42. 20709825 10.1164/rccm.201001-0107OC

[12] Inaba K, Inaba M, Romani N, Aya H, Deguchi M, et al (1992) Generation of large numbers of dendritic cells from mouse bone marrow cultures supplemented with granulocyte/macrophage colony-stimulating factor. J Exp Med 176: 1693–1702. 1460426 10.1084/jem.176.6.1693 PMC2119469

[13] R Development Core Team (2005). Vienna, Austria: R Foundation for Statistical Computing.

[14] Benjamini Y, Hochberg Y (1995) Controlling the false discovery rate: a practical and powerful approach to multiple testing.. Journal of the Royal Statistical Society Series B 57: 289–300.

[15] Yamaguchi KD, Ruderman DL, Croze E, Wagner TC, Velichko S, et al (2008) IFN-beta-regulated genes show abnormal expression in therapy-naive relapsing-remitting MS mononuclear cells: gene expression analysis employing all reported protein-protein interactions. J Neuroimmunol 195: 116–120. 18279974 10.1016/j.jneuroim.2007.12.007

[16] Suzuki K, Stenflo J, Dahlback B, Teodorsson B (1983) Inactivation of human coagulation factor V by activated protein C. J Biol Chem. 258: 1914–1920. 6687387

[17] Bajzar L, Morser J, Nesheim M (1996) TAFI, or plasma procarboxypeptidase B, couples the coagulation and fibrinolytic cascades through the thrombin-thrombomodulin complex. J Biol Chem 271: 16603–16608. 8663147 10.1074/jbc.271.28.16603

[18] Villadangos JA, Cardoso M, Steptoe RJ, van Berkel D, Pooley J, et al (2001) MHC class II expression is regulated in dendritic cells independently of invariant chain degradation. Immunity 14: 739–749. 11420044 10.1016/s1074-7613(01)00148-0

[19] Herrmann TL, Agrawal RS, Connolly SF, McCaffrey RL, Schlomann J, et al (2007) MHC Class II levels and intracellular localization in human dendritic cells are regulated by calmodulin kinase II. J Leukoc Biol 82: 686–699. 17586661 10.1189/jlb.0107045

[20] Chen T, Li Z, Jing T, Zhu W, Ge J, et al (2011) MicroRNA-146a regulates the maturation process and pro-inflammatory cytokine secretion by targeting CD40L in oxLDL-stimulated dendritic cells. FEBS Lett 585: 567–573. 21236257 10.1016/j.febslet.2011.01.010

[21] Dunand-Sauthier I, Santiago-Raber ML, Capponi L, Vejnar CE, Schaad O, et al (2011) Silencing of c-Fos expression by microRNA-155 is critical for dendritic cell maturation and function. Blood 117: 4490–4500. 21385848 10.1182/blood-2010-09-308064

[22] Turner ML, Schnorfeil FM, Brocker T (2011) MicroRNAs regulate dendritic cell differentiation and function. J Immunol 187: 3911–3917. 21969315 10.4049/jimmunol.1101137

[23] Niessen F, Schaffner F, Furlan-Freguia C, Pawlinski R, Bhattacharjee G, et al (2008) Dendritic cell PAR1-S1P3 signalling couples coagulation and inflammation. Nature 452: 654–658. 18305483 10.1038/nature06663

[24] Levy BD, Clish CB, Schmidt B, Gronert K, Serhan CN (2001) Lipid mediator class switching during acute inflammation: signals in resolution. Nat Immunol 2: 612–619. 11429545 10.1038/89759

[25] Serhan CN (2002) Lipoxins and aspirin-triggered 15-epi-lipoxin biosynthesis: an update and role in anti-inflammation and pro-resolution. Prostaglandins Other Lipid Mediat 68–69: 433–455. 10.1016/s0090-6980(02)00047-3 12432935

[26] Fukata Y, Oshiro N, Kinoshita N, Kawano Y, Matsuoka Y, et al (1999) Phosphorylation of adducin by Rho-kinase plays a crucial role in cell motility. J Cell Biol 145: 347–361. 10209029 10.1083/jcb.145.2.347 PMC2133101

[27] Larsson C (2006) Protein kinase C and the regulation of the actin cytoskeleton. Cell Signal 18: 276–284. 16109477 10.1016/j.cellsig.2005.07.010

[28] Torres-Aguilar H, Aguilar-Ruiz SR, Gonzalez-Perez G, Munguia R, Bajana S, et al (2010) Tolerogenic dendritic cells generated with different immunosuppressive cytokines induce antigen-specific anergy and regulatory properties in memory CD4+ T cells. J Immunol 184: 1765–1775. 20083662 10.4049/jimmunol.0902133

[29] Valastyan S, Weinberg RA (2011) Roles for microRNAs in the regulation of cell adhesion molecules. J Cell Sci 124: 999–1006. 21402873 10.1242/jcs.081513 PMC3056602

[30] Kim TD, Lee SU, Yun S, Sun HN, Lee SH, et al (2011) Human microRNA-27a* targets Prf1 and GzmB expression to regulate NK-cell cytotoxicity. Blood 118: 5476–5486. 21960590 10.1182/blood-2011-04-347526 PMC3217350

[31] Pedersen I, David M (2008) MicroRNAs in the immune response. Cytokine 43: 391–394. 18701320 10.1016/j.cyto.2008.07.016 PMC3642994

[32] Bai Y, Qian C, Qian L, Ma F, Hou J, et al (2012) Integrin CD11b negatively regulates TLR9-triggered dendritic cell cross-priming by upregulating microRNA-146a. J Immunol 188: 5293–5302. 22551553 10.4049/jimmunol.1102371

[33] Zhao KN, Masci PP, Lavin MF (2011) Disruption of spectrin-like cytoskeleton in differentiating keratinocytes by PKCdelta activation is associated with phosphorylated adducin. PLoS One 6: e28267. 22163289 10.1371/journal.pone.0028267 PMC3233558

[34] Habib GM, Shi ZZ, Cuevas AA, Lieberman MW (2003) Identification of two additional members of the membrane-bound dipeptidase family. FASEB J 17: 1313–1315. 12738806 10.1096/fj.02-0899fje

[35] Austen KF, Maekawa A, Kanaoka Y, Boyce JA (2009) The leukotriene E4 puzzle: finding the missing pieces and revealing the pathobiologic implications. J Allergy Clin Immunol 124: 406–414; quiz 415–406. 10.1016/j.jaci.2009.05.046 PMC2739263 19647860

[36] Spite M, Serhan CN (2010) Novel lipid mediators promote resolution of acute inflammation: impact of aspirin and statins. Circ Res 107: 1170–1184. 21071715 10.1161/CIRCRESAHA.110.223883 PMC3027152

[37] Maderna P, Godson C (2009) Lipoxins: resolutionary road. Br J Pharmacol 158: 947–959. 19785661 10.1111/j.1476-5381.2009.00386.x PMC2785518

[38] Barnig C, Cernadas M, Dutile S, Liu X, Perrella MA, et al (2013) Lipoxin a4 regulates natural killer cell and type 2 innate lymphoid cell activation in asthma. Sci Transl Med 5: 174ra126. 10.1126/scitranslmed.3004812 PMC3823369 23447017

[39] MacDonald KP, Munster DJ, Clark GJ, Dzionek A, Schmitz J, et al (2002) Characterization of human blood dendritic cell subsets. Blood 100: 4512–4520. 12393628 10.1182/blood-2001-11-0097

[40] Urban BC, Cordery D, Shafi MJ, Bull PC, Newbold CI, et al (2006) The frequency of BDCA3-positive dendritic cells is increased in the peripheral circulation of Kenyan children with severe malaria. Infect Immun 74: 6700–6706. 17000725 10.1128/IAI.00861-06 PMC1698077

