# Supplementary figures and images for: Differential Gene Expression in Thrombomodulin (TM; CD141)+ and TM− Dendritic Cell Subsets
Source: PLoS One. 2013 Aug 23;8(8):e72392. doi: 10.1371/journal.pone.0072392 (PMC3751914; doi:10.1371/journal.pone.0072392)

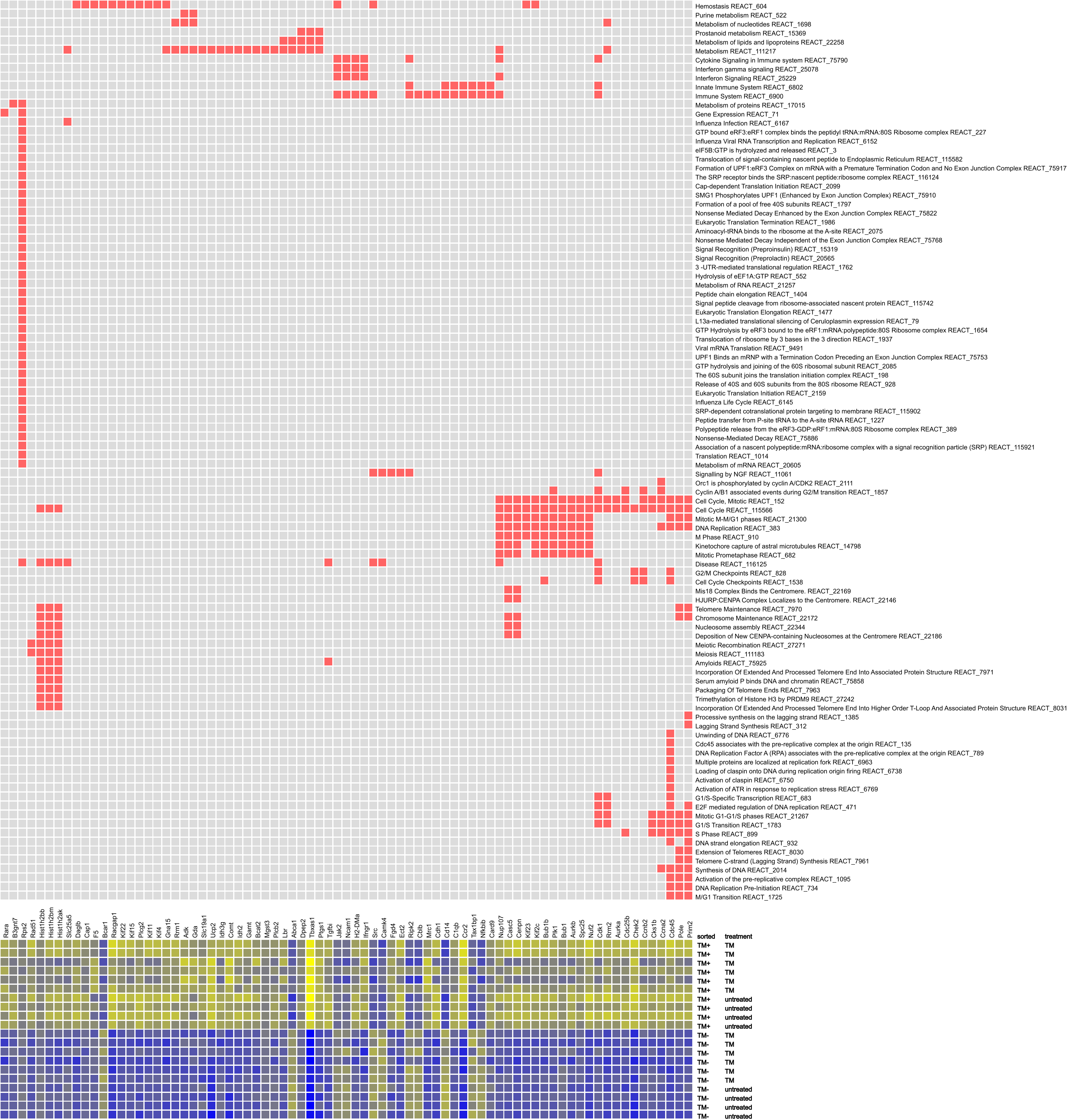

Supplement: Figure S1 — The top 100 gene sets identified as described in Materials and Methods are plot as a design structure matrix against the top 77 genes within those sets that changed significantly. A square is colored if a gene (column) belongs to a set (row). B. Heat map of the genes identified by the CERNO analysis of gene sets. Yellow is up-regulated, and blue is down-regulated. The depth of the colors is based on rescaled Z-values, with high values being yellow and low values blue. (PDF) [file pone.0072392.s001.pdf]
