# Supplementary material for: Differential Gene Expression in Thrombomodulin (TM; CD141)+ and TM− Dendritic Cell Subsets
Source: PLoS One. 2013 Aug 23;8(8):e72392. doi: 10.1371/journal.pone.0072392 (PMC3751914; doi:10.1371/journal.pone.0072392)
Supplement: Methods S1 — Ranking of transcripts using linear mixed effects model statistics. (DOCX) [file pone.0072392.s004.docx]

**Supplemental Methods S1: Ranking of transcripts using linear mixed effects model statistics**

Based on the experimental design, the data were analyzed using a linear mixed effects model for each transcript. The function lmer from the R (v2.11.1) library lme4 (v0.999375-41) was used for the model. The transcript expression value served as the dependent variable. We modeled the dependence of gene expression on the fixed effects TM sorting and TM treatment, and their interaction. The experiment was included as a random effects term to compensate for experiment-to-experiment variation in gene expression. The F-values for each fixed-effects term in each model were captured. Because we were interested in the specificity of the effect for the terms on the expression of each probe set, we calculated a proportion-weighted F (PWF) to rank probes sets. For k terms in the model, PWF of the kth term was calculated as PWF_k = F_k * F_k / sum_(i=1)^k F_i.

To characterize whether observed PWF values were large enough to be significant compared to random variation, we generated simulated PWF values by permutation of sample labels. The PWF values for each term in the model for 500 random label permutations were computed and results plotted and compared to PWF values calculated for the actual data.
